# Methods that shaped telomerase research

**DOI:** 10.1007/s10522-023-10073-8

**Published:** 2023-10-31

**Authors:** Louise Bartle, Raymund J. Wellinger

**Affiliations:** Department of Microbiology and Infectious Diseases, Faculty of Medicine and Health Sciences, Université de Sherbrooke, Applied Cancer Research Pavilion, 3201 rue Jean-Mignault, Sherbrooke, QC J1E 4K8 Canada

**Keywords:** Telomere, Telomerase, Telomerase RNA, Methods

## Abstract

Telomerase, the ribonucleoprotein (RNP) responsible for telomere maintenance, has a complex life. Complex in that it is made of multiple proteins and an RNA, and complex because it undergoes many changes, and passes through different cell compartments. As such, many methods have been developed to discover telomerase components, delve deep into understanding its structure and function and to figure out how telomerase biology ultimately relates to human health and disease. While some old gold-standard methods are still key for determining telomere length and measuring telomerase activity, new technologies are providing promising new ways to gain detailed information that we have never had access to before. Therefore, we thought it timely to briefly review the methods that have revealed information about the telomerase RNP and outline some of the remaining questions that could be answered using new methodology.

## Introduction

Methods to understand chromosome biology have evolved tremendously over the past 90 years. For the very ends of chromosomes, coined “telomeres” by Muller in 1938, philosophical and biochemical methods to understand their biological relevance have contributed to our overall knowledge of chromosome biology, cellular ageing, cancer and telomere-related disorders. Thanks to the development of many robust methods over the years, we now have a relatively good understanding of how telomeres are maintained and how the processes involved could impact health and disease, but many questions remain. Muller initially described the ends of chromosomes as repetitive or inactive “genes” (genes referring to blocks of nucleic acids, and not what we know as genes today) that showed resistance to X-ray damage (Muller 1938). At the same time, work by Barbara McClintock demonstrated the special nature of chromosome ends which apparently were able to heal after breakage (McClintock 1941). Yet, ends of any sorts are problematic when it comes to repair and initial thought experiments (also known as a *Gedankenexperimente*) by Olovnikov (1971) discussed the theory of “marginotomy”: the potential for detrimental losses of chromosome ends. In that paper, it was speculated that such losses should be overcome, at least in some cell types, and thus the telomeres may be extended by a hypothetical unidentified enzyme. Shortly after this, James Watson (1972) described the incomplete replication of chromosome ends based on work performed with T7 phage DNA, also alluding to the potential need for telomere extension. Since these stimulating initial interpretations of genetic, philosophical and biochemical experiments, a range of methods have helped unveil the proteins, RNA’s and cellular processes involved with telomere maintenance. The eventual discovery and characterisation of telomerase, the enzyme responsible for telomere extension, required a number of different methods to identify the proteins and RNA that make up this ribonucleoprotein (RNP), as well as to measure its catalytic activity. In this review, we discuss the major techniques used to study how telomerase maintains telomeres, including methods used to study DNA, RNA, proteins, whole cell/organism and in silico approaches, focusing on yeast and human research, with mention to foundational work in ciliates.

## Telomere length

Telomeres consist of repetitive tracts of DNA that, together with bound telomeric proteins, act as protective caps on chromosome ends (Wellinger and Zakian 2012; Bonnell et al. 2021; Lim and Cech 2021). Inability of the DNA replication machinery to duplicate both strands of DNA for the entire length of chromosomes results in telomere shortening and a G-rich 3’ overhang (Wellinger and Zakian 2012; de Lange 2018). In telomerase-positive cells (e.g. human stem cells, ciliates and yeast) telomeres can be extended and hence maintained, while in telomerase-negative cells (e.g. human somatic cells) telomere extension doesn’t happen and eventually cells enter replicative senescence. Ever since the initial discoveries of telomeres and telomerase there have been many methods to study telomeres at the molecular level. First off, telomere length measurements (i.e. measuring the length of DNA made up of telomeric terminal repeats), have been critical to understanding how telomerase maintains telomeres. The two main methods, Southern blotting (Southern 1975; Blackburn and Gall 1978) and fluorescence in situ hybridisation (FISH; Lansdorp et al. 1996), provide measurements of overall telomere length, with Southern blotting having been the gold-standard approach for decades. It was the first method developed for telomere length analysis, arising from initial work in ciliates (Blackburn and Gall 1978; Blackburn and Challoner 1984), and requires minimal training, aided by availability of commercial kits. Initially, the method required analyses of the DNA in question by digestion with Bal31, a nuclease that degrades dsDNA from the ends and hence shortens the terminal restriction fragments (TRFs) in Southern blots, which definitively showed the repetitive tracts were terminal DNA (Blackburn and Challoner 1984; Shampay et al. 1984; Walmsley and Petes 1985). For budding yeast, telomeric repeat tracts on individual telomeres in general are relatively short (~ 325 bp), and by using unique subtelomeric probes, Southern blotting can also be used for measuring DNA repeat length on specific individual chromosome ends. Such measurements gave rise to the dogma that all budding yeast telomeres carried about 325 bp (± 50 bp) of telomeric repeats (Walmsley et al. 1984; Walmsley and Petes 1985), despite the observation that at least the left telomere on chromosome 3 was much longer and did not fit that idea (Button and Astell 1986). In DNA derived from human cells, bulk telomere measurements by Southern TRF assay can reveal if telomeres are generally affected by cell type origin or culture conditions but cannot provide specific detail for individual chromosome ends. Due to the longer length and variation of human telomeres (5–15 kbp; de Lange et al. 1990; Canela et al. 2007), TRF bands create a long smear that makes it difficult to discern subtle changes in bulk telomere length. However, quantitative FISH (fluorescence in situ hybridisation) is an informative alternative method since human cell chromosome size and telomere lengths are discernible and quantifiable by fluorescence intensity (Lansdorp et al. 1996; for a detailed review see Lai et al. 2018). Though FISH can provide more detail than Southern blotting in human cells, it is restricted to mitotically dividing cell types and requires expertise in microscopy. Nevertheless, FISH has also been combined with flow cytometry (flow-FISH) to give quick results for telomere length and is able to be used in clinical applications (Baerlocher and Lansdorp 2003; Baerlocher et al. 2006). The benefit of flow-FISH includes availability of high-throughput analyses and comparatively fast results, though it is usually only used for a small subset of cell types. There are also PCR based approaches for human telomere length measurements, such as quantitative PCR (qPCR) and Single Telomere Length Analysis (STELA), that can be used for bulk or individual telomere analyses. qPCR and STELA have the advantages of being cost effective, faster to obtain results and requiring lower input DNA concentrations (Baird et al. 2003; Cawthon 2002, 2009). However, qPCR and STELA can have variability in both technical and biological replicates (Aubert et al. 2012; Aviv et al. 2011) and aren’t able to be easily compared between studies. In the case of STELA, to date it can only be applied to a few chromosomes that have unique subtelomeric regions or for telomeres less than 8 kb (Aubert et al. 2012). Given the technical challenges qPCR and STELA can pose, it remains questionable whether it is useful for broad applications and whether the information gained surpasses TRF assays.

**Fig. 1:**
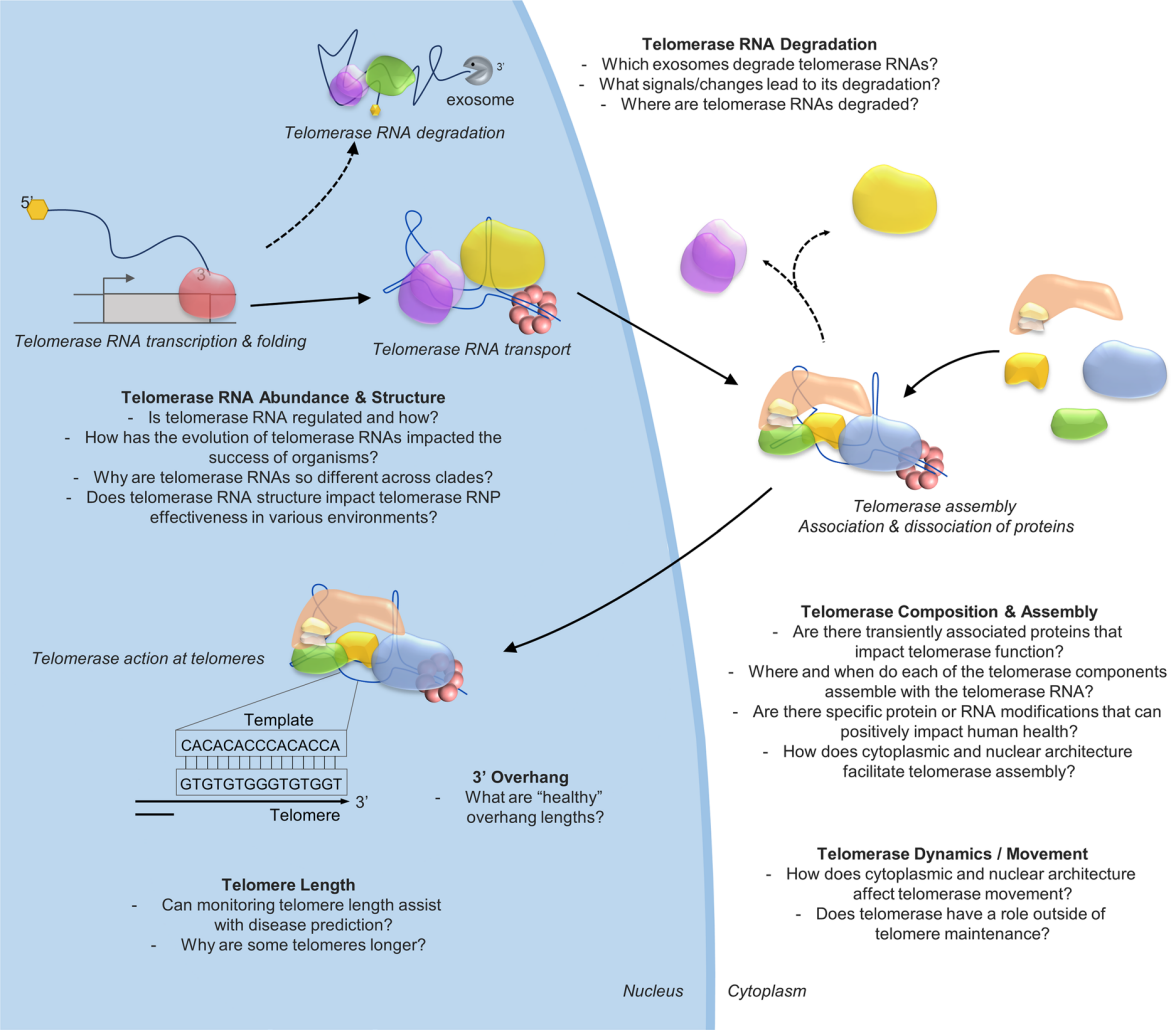
Generalised model of the telomerase lifecycle with some questions that remain to be answered. The questions are listed under the following sub-headings: Telomerase RNA Abundance & Structure; Telomerase RNA degradation; Telomerase Composition & Assembly; Telomerase Dynamics/Movement; 3’ Overhang and Telomere Length. The model is based on *Saccharomyces cerevisiae* telomerase processes and includes some species-specific variations of telomerase RNP processes

Despite the robustness and repeatability of Southern blotting and FISH for overall telomere length, neither provide specific base-pair length determination of all individual telomeres, and potentially cannot detect missing telomeres. To tackle these problems, recent major advancements in long-read sequencing technology (PacBio; Eid et al. 2009 or Oxford Nanopore sequencing; Jain et al. 2016) are being exploited to determine individual telomere lengths in both yeast (Sholes et al. 2022) and humans (Tham et al. 2023). Unlike Southern blotting and FISH protocols, sequencing can be more easily applied to large sample sizes and has been successfully used to determine yeast telomere length for over 1000 isolates (D’Angiolo et al. 2023). Unlike other methods such as STELA or flow-FISH, telomere sequencing could also be used for a much larger range of cell types and tissues, overcoming some of the limitations with regard to cell division status or sample type. While this new telomere sequencing approach is very promising, there remain issues with alignment of repetitive telomeric sequences and general concerns related to individuality vs artefact. Though sequencing individual telomeres has great potential for human telomere-related disease diagnosis, much work is still required to create and implement robust, efficient and user-friendly DNA extraction, sequencing and analysis protocols for widespread use and for various cell types. Once these challenges are overcome, sequencing may be able to provide new information regarding how telomere length patterns in various cell types or through ancestry could predict probabilities of telomere-associated disease and cancers. Telomere sequencing could also be used to find out why some chromosome ends are longer (is it related to the location of specific genes, for example mating type loci or essential genes?), whether specific telomere lengths are impacted by ageing or general health and even potentially be used to find nucleotide-level genomic changes that could increase telomerase attraction to telomeres (Fig. 1).

Alongside telomere length, early on there was the critical observation that telomeres are left with a single stranded 3’ overhang (also known as a G-tail; Klobutcher et al. 1981; Henderson and Blackburn 1989; Wellinger et al. 1992). This overhang is required for telomerase to bind and extend telomeres, and in human cells deficient in telomerase subunits, the overhang length is impacted (Chai et al. 2006). In order to analyse G-tails, an in-gel hybridisation assay was developed that could be used to detect and quantify those single stranded telomere overhangs (Dionne and Wellinger 1996). While this method requires some expertise, it undoubtedly provides a clear way to study 3’ G-tails, and clarified the telomerase-independent generation of 3’ telomere overhangs as well as the timing of telomerase-mediated telomere extension (Wellinger et al. 1993; Dionne and Wellinger 1996). In-gels are now standardly used for characterisation of alternative lengthening of telomeres (ALT) mechanisms in human cell lines, and remain the gold- standard for single-strand analysis. Later, ligation-based methods such as ligation mediated primer extension (Jacob et al. 2001) and telomeric repeat oligonucleotide ligation assay (T-OLA; Cimino-Reale et al. 2001) provided more precise measurement of 3’ overhangs in ciliate and human cells, helping determine the frequency of short G-tails (less than 90 nt) which was underestimated by other methods such as electron microscopy. The double-strand specific nuclease (DSN) method was developed to also detect short overhangs of minimally 12 nucleotides in length (Zhao et al. 2008). This approach has been successfully used for human cell lines and provides a way to specifically assess the impact of telomerase dysfunction on overhang length.

All methods for G-tail analysis are labour intensive and as such aren’t suitable for high-throughput analyses. Despite the complexity and intensive nature of 3’ overhang analyses, they are not limited to certain cell types and therefore can be used to study a number of disease phenotypes or be used to understand healthy ageing, possibly answering: what G-tail lengths are deemed “healthy” during each life stage? (Fig. 1) Sequencing could be an alternative method for large-scale analysis of 3’ overhangs, since long-read technology offers sequencing to the very end of chromosomes and with improved sequencing quality and bioinformatics tools, the two cognate strands could be specifically aligned. Performing such strand-specific alignment of sequencing reads could detail overhang length in addition to overall telomere length and hence provide a well-rounded view of telomere metrics for any and every cell type. Eventually this may contribute to individual and cell-type specific medical analysis and treatments. But until high-throughput methods can be devised for telomere overhangs, there remain questions pertaining to how 3’ overhangs are implicated in telomere-disorders, and the overall impacts of overhang length on general health.

## Telomerase activity

The initial findings of telomeres naturally led to theories that there would be a process for telomere repeat addition. Biochemistry aided the discovery that telomeric repeat addition was an enzymatic process; findings that were found worthy of the Nobel prize (Greider and Blackburn 1985, 1987). Once it was clear that telomeric repeats were maintained by a reverse transcriptase type enzyme, dubbed telomerase, it became crucial to analyse all aspects of telomerase biology, from its generation to its loss. Assessing catalytic telomerase activity in various situations yielded specific information about proteins and RNA that are required for telomerase functionality. In yeast and human cells, after a certain enrichment of telomerase, this catalytic activity can be measured using adaptations of a direct radionucleotide addition assay (Morin 1989; Cohn and Blackburn 1995) that was originally created for ciliate cell extracts (Greider and Blackburn 1985, 1987). For more sensitive detection, a PCR-based telomerase repeated amplification protocol (TRAP) assay was later developed (Kim et al. 1994). TRAP assays have been modified to use non-radioactive fluorescent-labelled nucleotides to improve safety and accessibility (Herbert et al. 2006), or to include amplification steps to optimise for different cell types (Blasco et al. 1997). Since testing telomerase activity remains incredibly useful, there are now commercial kits that make this technique available for clinical use. While the above assays, which target enzymatic activity, have helped to identify proteins that are required for telomerase directly, they do not inform on assembly or in vivo action at telomeres. Telomerase assays may be used for individual cell types; however, isolation and purification of telomerase can be difficult, and is therefore not suitable for large scale applications.

While traditional telomerase assays do provide information about bulk telomerase activity from whole cell extracts, more recent methods for measuring telomerase activity can assess the RNP at a single molecule level. This level of detail is necessary to identify factors that could affect telomerase activity in specific ways: such as substrate binding, nucleotide addition processivity and repeat addition processivity. Incredibly detailed observations of telomerase catalysis on telomeric substrates have been achieved using optical tweezers (Patrick et al. 2020). Offering the highest resolution thus far, optical tweezers is an in vitro approach where individual RNPs can be tested for their ability to perform telomeric repeat extension. Such methods offer the same information as classical TRAP and radionucleotide addition assays, the catalytic activity of telomerase, but also have confirmed a secondary DNA binding site on human telomerase, and nucleotide-scale detail on the stepwise nucleotide addition process (Patrick et al. 2020). Understanding telomerase activity at this detailed scale could provide insights into telomerase dysfunction that occurs during nucleotide or repeat processivity and provide even more information regarding certain telomeropathies. As discussed by Opresko and Shay (2017), telomere-related syndromes will continue to be discovered and diagnosed as new tools become available for clinically relevant measurements of telomere maintenance.

## Telomerase RNAs

An essential component of telomerase is a templating RNA that provides the sequence for telomere extension. As such, many methods have been used to measure telomerase RNA abundance, shape, processing and location inside cells. Telomerase RNA abundance is of particular interest since the RNA is the limiting factor for telomerase in yeast (Mozdy and Cech 2006; Bajon et al. 2015) and maintained as a steady equilibrium in human cells (Xi and Cech 2014). Additionally, higher levels of telomerase RNA are linked to aberrant effects in both humans (linked to cancers; Pruzan et al. 1990; Avilion et al. 1996) and yeast cells (causing telomere shortening; Singer and Gottschling 1994), indicating that telomerase RNA concentration needs to be restricted for healthy cell function. It therefore seems worthy to consider methods for analyses of telomerase RNAs in some detail.

Human and yeast telomerase RNAs have been successfully quantified by Northern blot and qPCR, revealing that there are only relatively few molecules at any one time (Avilion et al. 1996; Lingner et al. 1997b; Mozdy and Cech 2006; Noël et al. 2012; Tseng et al. 2015). In addition to these assays, single-molecule FISH has been used for “ground-truth” measurement of telomerase RNA abundance, while also giving subcellular localisation information (Zhu et al. 2004; Gallardo et al. 2008; Bajon et al. 2015; Vasianovich et al. 2020). The use of fluorescence resonance energy transfer (FRET), for precise measurement of human telomerase RNA abundance in cells may provide an alternative to FISH (Zhang et al. 2022), in addition to being used for proximity interactions between telomerase RNA and proteins or telomeres. Collectively these assays have contributed to our understanding that maintaining telomerase RNA abundance is pivotal to telomerase function and overall cell health.

In terms of the RNA’s secondary structure, alignment and folding algorithms using phylogenetics were used initially for predicting the structure of ciliate telomerase RNAs (Romero and Blackburn 1991; McCormick-Graham and Romero 1995) and later vertebrates, including human telomerase RNA’s (Chen et al. 2000; Chen and Greider 2004). The yeast phylogenetic predictions (Zappulla and Cech 2004; Dandjinou et al. 2004) were experimentally supported by site specific RNAse H cleavage assays (Dandjinou et al. 2004) and dimethylsulfate sensitivity assays (Förstemann and Lingner 2005). Later selective 2′-hydroxyl acylation analysed by primer extension (SHAPE; Wilkinson et al. 2006; Laterreur et al. 2018) helped increase secondary structure resolution of certain areas of the yeast telomerase RNA. These secondary structures gave more insight into the divergence of telomerase RNAs across kingdoms and provided a starting point for understanding overall telomerase RNP configuration. More specific detail has been achieved using nuclear magnetic resonance (NMR) that revealed folding of the telomerase RNA pseudo-knot (Theimer et al. 2005).

Further to overall prediction of RNA structure, methods such as northern blot and qPCR have revealed the presence of longer, unprocessed forms of telomerase RNAs (Seto et al. 1999; Noël et al. 2012; Tseng et al. 2015) with 3’ rapid amplification of cDNA ends (RACE) coupled with deep sequencing further identifying heterogeneity of 3’ ends of human telomerase RNAs (Goldfarb and Cech 2013). For the 5’ end, TMG-RNA immunoprecipitation and anti-TMG immunoblotting revealed a trimethyl guanosine cap on human and yeast telomerase RNAs (Seto et al. 1999; Jády et al. 2004).

However, there remains missing information about whether telomerase RNA levels fluctuate throughout the cell cycle (Fig. 1), given the broad distribution of telomerase RNA quantity from a population of cells (Vasianovich et al. 2020). This may be answered using live-cell microscopy and long-term observation of single cells, made possible by use of microfluidics devices in combination with fluorescent RNA aptamers and proteins (as discussed later). Accurate, rapid and detailed telomerase RNA abundance measurements could contribute to our overall understanding of ageing, and when combined with telomere length analysis and telomerase activity, could help manage healthy ageing or predict tissue-specific disease onset.

In addition to telomerase RNA abundance, the half-life of telomerase RNAs has been measured using transcription arrest assays. This method identified the uncharacteristically long stability of telomerase RNA in human (Yi et al. 1999) and yeast cells (Larose et al. 2007), however the method ultimately kills cells and in the case of yeast telomerase RNA, it could only provide a half-life estimate of  > 1 h (Larose et al. 2007). To date no further methods have been used to gain more specific half-life information for yeast and human telomerase RNAs, but figuring out the reason for such longevity could give invaluable information about telomerase biogenesis, and provide new avenues for treating telomerase RNA disorders. There also remains the question: what mechanisms are involved with telomerase RNA degradation? (Fig. 1) Using gene technology that can selectively target “old” telomerase RNAs without affecting cell function will be useful for studying these long-lived RNAs. Photo-switchable fluorescent proteins like Dendra2 (Gurskaya et al. 2006; Auboiron et al. 2021) could be used in conjunction with RNA–protein binding systems (i.e. MS2-stem-loop:MS2-coat protein binding; Bertrand et al. 1998; Gallardo et al. 2008; Laprade et al. 2020) or by inducible RNA modification as in Vasianovich et al. (2020), enabling specific and long-term analysis of old telomerase RNAs. On the other hand, telomerase RNA degradation could be studied using deletion of exonucleases that target RNAs, or using an inducible protein degron system (Holland et al. 2012) to determine if loss of particular telomerase components leads to telomerase RNA degradation. Tools to answer questions about telomerase RNA longevity could also uncover new details of the overall telomerase RNA lifecycle.

As mentioned above, telomerase RNAs are dynamic and ever-changing molecules, undergoing 3’ and 5’-end processing steps, association with transport proteins, and assembly with telomerase components before acting at telomeres. Using microscopy-based methods, telomerase RNAs have been found to localise in different cellular compartments to undergo these maturation processes (Gallardo et al. 2008, 2011; Nguyen et al. 2015; Vasianovich et al. 2020; Buemi et al. 2022). FISH with telomerase RNA specific fluorescent probes yielded details about cell compartment distribution of the RNA, and in combination with recombination-based molecular tools, was used to identify new components involved in early-RNA shuttling (Zhu et al. 2004; Gallardo et al. 2008; Vasianovich et al. 2020). Localisation of telomerase RNA with proteins has been achieved using immunofluorescence, that helped to define when the RNA was being processed, shuttled or acting at telomeres (Tomlinson et al. 2006; Cristofari et al. 2007; Cusanelli et al. 2013). Likewise, fluorescent labelling of specific telomeres helped identify the timing of telomere extension during the cell cycle (Tomlinson et al. 2006). Telomerase RNA dynamics in cells were more accurately documented using live-cell approaches. For both human and yeast telomerase RNA, coupling of MS2-tagged RNA with GFP-MCP has revealed the location of telomerase RNAs within the cell and association with telomeres at various points of the cell cycle, providing great detail about the overall telomerase RNA lifecycle (Gallardo et al. 2011; Laprade et al. 2020). The microscopy-based methods for investigating telomerase RNA dynamics are time intensive, small-scale analyses that require expertise and equipment that isn’t widely available. However, visual data produced using microscopy can help address questions regarding telomerase RNA localisation, clustering and action at telomeres that may not be easily determined using biochemical methods alone. Indeed, knowing specific details of telomerase RNA processing can provide avenues for disease treatments. One such process, 5’ capping of telomerase RNAs by Tgs1 in Cajal bodies (Verheggen et al. 2002) has been identified as a potential therapeutic target, where alleviating Tgs1 capping of telomerase RNAs and inhibiting telomerase action could treat cancers, or increasing Tgs1-telomerase RNA functionality could be used for treating premature ageing disorders (Buemi et al. 2022).

All together fixed and live-cell methods have provided incredible details about telomerase RNA biogenesis, but there remains opportunity for better resolution. New tools for RNA tagging and higher resolution microscopy that can offer even more detailed observations of cellular processes continue to be developed. Brighter fluorescent proteins (Lambert et al. 2020), direct RNA-fluorophore binding like mango and peach RNA aptamers (Cawte et al. 2020; Kong et al. 2021), and microfluidics devices (Gao et al. 2020; Anggraini et al. 2022), to name just a few, could all provide greater and more specific detail of telomerase dynamics that could never have been done before. For new tools that utilise dyes, in the case of yeast, it requires some optimisation since the yeast cell wall is a great barrier to foreign molecules. Though this challenge may be mitigated by mutating multidrug transport pumps, a successful approach used for HaloTag dyes (Podh et al. 2022). As for microfluidics devices, they require particular and costly set-ups, however the detail gained for long-term imaging is incomparable. Microfluidics devices can include options for changing media or adding drugs, dyes and nutrients that could be used in conjunction with inducible molecular tools. The constant improvement in microscopes will allow greater resolution and clarity for small cell (in the case of yeast), intracellular compartment, and long-term imaging, solidifying these methods as powerful tools for telomerase research.

## Telomerase proteins

The full composition of telomerase in yeast and human cells is still under investigation because it comprises many different proteins, some of them being part of telomerase only transiently. Before we delve into the methods for telomerase protein analysis, we should get to know the key players. Telomerase minimally requires a catalytic reverse transcriptase, known as Est2 in yeast and TERT in humans, together with telomerase RNA. Surrounding the catalytic core are a number of proteins that assemble with the RNA scaffold, or between telomerase subunits, some of which are necessary for subcellular localisation and association at telomeres. For yeast, these include Est1, Est3, Pop1 Pop6, Pop7, the yKu70/80 dimer and 3’ Sm7 ring (for a review see Bartle et al. 2022). For human telomerase they comprise TCAB1, NHP2, GAR1, NOP10 and dyskerin (Nguyen et al. 2018). Many techniques have been used to identify, characterise and further study proteins that associate with- and impact telomerase formation and subsequent interaction with telomeres. Immuno co-purification of proteins has helped identify Est1, Est2, Est3, Pop1, Pop6, Pop7 and Sm7 proteins in yeast and TERT, NHP2, NOP10, dyskerin and GAR1 in human telomerase RNPs (Harrington et al. 1997a, 1997b; Lingner et al. 1997a; Mitchell et al. 1999; Seto et al. 1999; Dragon et al. 2000; Zhou et al. 2000; Mitchell and Collins 2000; Pogačić et al. 2000; Hughes et al. 2000; Fisher et al. 2004; Lemieux et al. 2016). This method relies on epitope-tagging proteins of interest, purifying them from crude extract and then usually using reverse-transcriptase quantitative PCR (RT-qPCR) or Northern blot to determine the presence of telomerase RNA. Using immunoprecipitation, it has been possible to identify proteins either by using indirect pull-down of the RNA using the MS2-MCP system (Laterreur et al. 2018), by mass-spectroscopy as a de-novo approach to identify associated proteins (Lemieux et al. 2016), and by simultaneous detection of epitope tagged proteins (co-immunoprecipitation; Witkin and Collins 2004; Fu and Collins 2007; Takai et al. 2010; Laterreur et al. 2018). These biochemical assays provide strong evidence for telomerase-associated proteins when also coupled with telomerase activity assays.

## Structure function studies of the telomerase RNP

Collectively the above-mentioned methods yielded intricate details of telomerase RNPs, but none approached the overall resolution achieved by cryogenic electron microscopy (cryo-EM). Initially cryo-EM provided a 3D structure for ciliate telomerase (Jiang et al. 2015), revealing new subunits and the configuration of telomerase RNA within the catalytic core. More recently the use of cryo-EM has been used to further define the 3D structure of human telomerase, revealing telomerase RNP conformation at sub-nanometre resolution (Nguyen et al. 2018; Liu et al. 2022). This further defined interaction sites between the RNA and protein subunits, and even uncovered new pieces in the puzzle of telomerase architecture. As with human telomerase, we anticipate that the 3D structure of yeast telomerase with the telomerase RNA will also be solved using cryo-EM and therefore potentially provide new details about that RNP. Uncovering the intricate details of telomerase structure has provided more detail regarding the zipper mechanism of telomere extension (Wan et al. 2021), and telomerase-associated disorders. For example, a number of amino acid mutations in TERT lead to disease, and through cryo-EM it was revealed that these mutations are often sites bridging between TERT and the telomerase RNA (Liu et al. 2022).

Cryo-EM, unlike other protein structure techniques, does not require crystals. Images of multiple angles of the protein complex are merged, and using specialised software the atomic resolution, 3D models of proteins and their substrates are created (Weissenberger et al. 2021). Although cryo-EM achieves unbelievable resolution of RNPs, it represents the average structure from many individual purified complexes. The process of obtaining structures from cryo-EM can lead to exclusion of minority forms of the telomerase RNA (such as 3’ extended and unprocessed forms), or transient proteins that interact with the telomerase RNP. It is also difficult to differentiate RNPs that are broken (due to methodological process) versus RNPs that are in their immature form. Therefore, other more specific methods are still needed to assess the full population of telomerase RNPs. Still, detailing the structure of telomerase and telomerase RNAs can give valuable information about telomerase-disorders that arise from mutations in telomerase RNA and proteins.

Protein purification-reliant methods such as pull-down assays or cryo-EM have been very useful for identifying subunits that make-up telomerase RNPs and for studying telomerase-genomic loci interactions. However, these methods usually rely on bulk protein extraction from cells, which do not provide subcellular detail and may lead to underrepresentation or inability to detect transient protein interactions. Combining pull-down assays or cryo-EM with cell fractionation may offer more specific details of compartment-specific telomerase RNP assembly or processing, while single cell analysis may be able to help catch transient interactions and visualise telomerase movement.

Given that telomerase is dynamic, shuttling between nuclear and cytoplasmic compartments, microscopy has aided our understanding of the timing of telomerase action. Using HaloTags, human telomerase has been directly tracked to see when it acts at telomeres, revealing instances of short and long associations and unveiling the transient nature of telomerase -telomere association (Schmidt et al. 2016), as well as the specific subcellular location and assembly of telomerase with telomerase RNA (Klump et al. 2023). Other promising tools for telomerase microscopy are Mango and Peach RNA aptamers (Cawte et al. 2020; Kong et al. 2021). Direct binding of fluorescent biotin to telomerase RNA would give a new way to directly track the RNA and telomerase as a whole. Additionally, Mango and Peach RNA aptamers can be used for protein enrichment assays (Panchapakesan et al. 2017). In this way, experiments could have both microscopy and protein assays using the same tool, and therefore a direct correlation between experiments. Pin-pointing the when and where of telomerase association at telomeres has helped provide mechanistic details about how telomerase is retained on telomeres to perform its function (Schmidt et al. 2018; Laprade et al. 2020). Our current understanding of the dynamic nature of telomerase raises question as to when telomerase components assemble. For now, it remains unclear when each protein subunit comes together on the telomerase RNA scaffold, but utilising immunoprecipitation, biotinylation and proximity assays in single cell analyses may provide us these details. There is still more to be learned about telomerase dynamics in different cell types, and there remains opportunity to discover how this RNP performs over a lifetime in various cells and tissues.

## Going beyond the bench: in silico approaches

Alongside laboratory techniques are in silico approaches. The increasing availability of high-powered computing, genome databases, prediction tools and specialised software have propelled the telomere research field into a new age. Modelling and other complex statistical analyses are becoming increasingly useful for determining kinetics and associations that may otherwise be missed solely by biochemical approaches. Not only is accessibility to new sequencing technologies enabling new insight to expression changes, but it has helped with prediction of protein folding (e.g. AlphaFold; Jumper et al. 2021; Varadi et al. 2022), and notably the determination of telomerase structure after cryo-EM (Nguyen et al. 2018). Tools for RNA folding predictions based on known energies and attractions between base pairs help scientists visualise RNA structures, ultimately aiding our overall understanding of protein-RNA interactions (Zuker 1989, 2003; Romero and Blackburn 1991; Thompson et al. 1997; Chen et al. 2000; Tzfati et al. 2003; Dandjinou et al. 2004). Although the structures determined by predictive tools can, and should, be tested and confirmed by laboratory-based methods because parameters for in silico folding are easily manipulated.

Further to predictive RNA and protein structure tools, there are great advancements in modelling of biological systems. The combination of complex statistical, mathematical and biological models leads to new insights of cellular processes that, in the past, could only be speculated. Modelling of biological systems has been used to understand replicative senescence (Martin et al. 2021), generating information that may not arise from laboratory protocols alone. Modelling has also proven very useful for understanding telomerase dynamics in cells (Laprade et al. 2020), and provides a way to derive trends from complex and multifactorial data. Other than modelling, analysis of large-scale and complex sequencing data has been made possible by advances in bioinformatics (Sholes et al. 2022; Tham et al. 2023; D’Angiolo et al. 2023), utilising high-powered computing and creating specific software or bioinformatic tools to meet the demands of our research questions.

With great strides always occurring in laboratory and computer sciences, there are great tools that will be developed to enable the next generation of scientists to delve even deeper into telomerase structure, function and biogenesis in ways that previous researchers could not even imagine.

## Conclusions

A broad range of biochemical and computer-reliant techniques have led us to our current understanding of the complex characteristics and dynamics of telomerase. While gold standard methods such as telomerase activity and telomere length assays remain useful tools for initial phenotypic confirmation in experiments, use of latest generation technologies like long-read sequencing, cryo-EM and modelling have led to more precise and more detailed views of the structure and function of this important RNP. Even though we have more technology than ever before to study telomerase, human cell-line and population studies encounter particular problems when it comes to studying telomeres and/or the telomerase RNP. Long-term analyses in human cell-lines are restricted to immortalised cell types which may display telomere phenotypes that arise from issues with the cells and therefore may not reflect what would occur in normal cells. At a larger scale, human studies rely on blood samples for studying telomeres in a population and would therefore miss trends in telomere or RNP changes that are specific to other tissues. This highlights that there is much more to learn about telomeres and the telomerase RNP over the duration of human lifespans, from population scale down to single cells.

New technologies and procedures are required to answer unsolved questions about telomerase and telomeres (Fig. 1), including whether the 3’ telomere overhang is implicated in health and disease; how is the telomerase RNA degraded; when and where do telomerase proteins assemble; does telomerase RNA fluctuation occur and how is it regulated? Do protein and RNA modifications play a role in disease and general health? How has the evolution of telomerase RNAs impacted the success of organisms, and why are telomerase RNAs so different across clades? Do these differences in telomerase RNA structure correlate to its effectiveness in telomerase RNPs? Does telomerase have a role outside of telomere maintenance, and what are the molecular mechanisms involved or the biological implications of this? And since we know that cellular compartments are not biochemically homogeneous, how does nuclear and cytoplasmic organisation and architecture affect telomerase assembly, movement and function?

With increasing precision and power, new technologies can help answer these questions and continue to unveil the complex mechanisms of telomerase.

## Data Availability

Not applicable.
